# Tendon Is Covered by a Basement Membrane Epithelium That Is Required for Cell Retention and the Prevention of Adhesion Formation

**DOI:** 10.1371/journal.pone.0016337

**Published:** 2011-01-26

**Authors:** Susan H. Taylor, Sarah Al-Youha, Tom Van Agtmael, Yinhui Lu, Jason Wong, Duncan A. McGrouther, Karl E. Kadler

**Affiliations:** 1 Wellcome Trust Centre for Cell-Matrix Research, Faculty of Life Sciences, University of Manchester, Manchester, United Kingdom; 2 Plastic and Reconstructive Surgery Research, Faculty of Medicine and Human Sciences, University of Manchester, Manchester, United Kingdom; 3 Institute of Cardiovascular and Medical Sciences, College of Medical, Veterinary and Life Sciences, University of Glasgow, Glasgow, United Kingdom; Ohio State University, United States of America

## Abstract

The ability of tendons to glide smoothly during muscle contraction is impaired after injury by fibrous adhesions that form between the damaged tendon surface and surrounding tissues. To understand how adhesions form we incubated excised tendons in fibrin gels (to mimic the homeostatic environment at the injury site) and assessed cell migration. We noticed cells exiting the tendon from only the cut ends. Furthermore, treatment of the tendon with trypsin resulted in cell extravagation from the shaft of the tendons. Electron microscopy and immunolocalisation studies showed that the tendons are covered by a novel cell layer in which a collagen type IV/laminin basement membrane (BM) overlies a keratinised epithelium. PCR and western blot analyses confirmed the expression of laminin β1 in surface cells, only. To evaluate the cell retentive properties of the BM *in vivo* we examined the tendons of the *Col4a1^+/Svc^* mouse that is heterozygous for a G-to-A transition in the *Col4a1* gene that produces a G1064D substitution in the α1(IV) chain of collagen IV. The flexor tendons had a discontinuous BM, developed fibrous adhesions with overlying tissues, and were acellular at sites of adhesion formation. In further experiments, tenotomy of wild-type mice resulted in expression of laminin throughout the adhesion. In conclusion, we show the existence of a novel tendon BM-epithelium that is required to prevent adhesion formation. The *Col4a1^+/Svc^* mouse is an effective animal model for studying adhesion formation because of the presence of a structurally-defective collagen type IV-containing BM.

## Introduction

Tendons are fibrous tissues that provide attachment of muscles to bone. The repetitive contraction and relaxation of muscles requires that tendons glide smoothly past adjacent tissues. The properties of the tendon surface that enable gliding and define the boundaries of the tissue are poorly understood. However, following tendon damage as a result of trauma, surgery, infection, and inflammatory disease, abnormal fibrous adhesions form between the tendon surface and overlying tissues [1], [2]. These adhesions are a ‘hidden disease’ with no effective treatment or cure [3]. Tendon injuries and adhesions are common in children, athletes, the aged, and manual workers, resulting in pain and disability. As summarised by Butler and co-workers, more than 32 million traumatic and repetitive motion injuries to tendons and ligaments occur annually in the USA [4]. Surgery usually provides the patient with the best chance of recovery but is only partially successful because of the interactive problems of adhesions leading to impaired movement through inhibition of normal tendon gliding [5]. The mechanism of adhesion formation is unknown. Current hypotheses include blood vessel in-growth, inflammation, cellular proliferation, synthesis of collagen and new extracellular matrix, and vascularisation (see [6] for review). A common theme, however, is the occurrence of adhesions at the site of injury of the tendon surface where fibrin clots form during haemostasis. In this study we aimed to shed light on how tendon adhesions are formed.

Cavities and structures within the body are covered by epithelial, endothelial or mesothelial cells that encapsulate and compartmentalise tissues thus allowing specialized organ function and movement of nutrients and waste products at cell-air and cell-liquid interfaces. These surface-located cells reside on basement membranes (BMs), which are sheet-like protein structures that are essential for cell differentiation, survival, adhesion, proliferation and migration as well as tissue scaffolding and filtration (for review see [7]). BMs comprise a variety of specialized macromolecules including laminins that provide survival signals to epithelial and endothelial cells [8] and are essential for BM formation [9]. The rod-like molecules of collagen IV link together to form a porous scaffold that provides mechanical stability and supports the filtration properties of BMs. Nidogens form protein complexes between laminin and collagen IV. BMs also contain the heparan sulfate proteoglycans perlecan, agrin and collagen XVIII, which have the capacity to bind cytokines and growth factors via their glycosaminoglycan side chains (for review see [10]).

## Materials and Methods

### Reagents

DMEM (Dulbecco's Modified Eagle's Medium, high glucose), L-ascorbic acid 2-phosphate, fetal calf serum (FCS), phosphate buffered saline (PBS), rabbit anti-ZO-1 antibody and goat/donkey anti-rabbit/mouse Cy3 secondary antibodies were purchased from Invitrogen, UK. Rabbit anti-claudin-1 antibody was from Zymed Laboratories and rabbit polyclonal antibodies to keratin 1 and keratin-10 were purchased from Abcam. The laminin antibody was a kind gift from Dr. Ulrike Mayer and the H22, H31, H69 antibodies against α2(IV) collagen chain, α3(IV) collagen chain and α6(IV) collagen chain, were from Dr. Yoshikazu Sadu. The nidogen 1 and perlecan antibodies were kindly donated by Dr. Rupert Timpl and Dr. Takako Sasaki, respectively. Fibrinogen, thrombin, Tween20, bovine serum albumin (BSA), proteinase K, toluidine blue and anti-rabbit laminin antibody were from Sigma Aldrich, UK. Calcein AM (Calbiochem, NJ, USA) was prepared as 10 mM solution in 0.1% v/v DMSO. Goat anti-mouse/rabbit Alexa488 secondary antibodies and Vectashield mounting medium with DAPI (4′,6-Diamidino-2-phenylindole dihydrochloride) were purchased from Vector labs, Peterborough, UK. Tissue-Tek mounting medium was bought from Fisher Scientific, Loughbourough, UK. TaqMan reverse transcriptase (RT) polymerase and BigDye Terminator v3.1 cycle sequencing kit were supplied by Applied Biosystems. The Mikro-Dismembrator was from Braun Biotech International. PCR primers (chicken) β-actin forward: GCCACAGCTGCCTCTAGCTCT; β-actin reverse: CAGCACTGTGTTGGCATACAG. Laminin β1 forward: CCCAGTACTCCTTGGTGGAA; laminin β1 reverse: ACACCGGTATCTCTGGAACG.

### Ethical statement

All animal procedures were approved by the Local Ethical Review Process at the University of Manchester and University of Glasgow and complied with the relevant licenses (University of Manchester PPL 40/2734, PIL 60/9273 and University of Glasgow PPL 60/4132, PIL 60/10027) approved by the UK Home Office on the Care and Use of Laboratory Animals.

### Tendon culture in a fibrin gel

Tendons were carefully removed by dissection to minimise damage to the surface. These were encased within a fibrin gel (20 mg/mL fibrinogen and 200 U/mL thrombin (Sigma, St Louis, MO, USA)) and the tendons were cultured in DMEM medium supplemented with L-ascorbic acid 2-phosphate (200 µm), penicillin/streptomycin (1%) and 10% fetal calf serum for 5 days at 37°C in a humid atmosphere containing 5% CO_2_. The tendon-fibrin gel was incubated in a 6-well plate pre-coated with Sylgard [11]. In experiments in which tendons were proteolytically damaged by trypsin, the tendons were incubated at 37°C for 15 min in 0.25% trypsin in Hanks Balanced Salt Solution (HBSS, pH 7.5). Live cell imaging was performed on a Leica SP2 Inverted Microscope connected to a personal computer with Leica Confocal Software (Leica Microsystems, Heidelberg, Germany). A heated environmental chamber maintained the tendons at 37°C and with humid atmosphere of 95% air/5% CO_2_.

### Immunofluorescence

Cryosections of chick tendon (10 µm) or mouse flexor tendon (8 µm) were fixed in 100% acetone at −20°C for 10 min and blocked with 10% normal goat serum in PBST (10 mM sodium phosphate, 140 mM NaCl, pH 7.4, 0.1% Tween 20) for 1 hr. Sections were incubated in primary antibody diluted in 5% BSA in PBST overnight at 4°C, washed for 5×10 min with PBST and incubated with goat or donkey anti rabbit/mouse-Cy3 (1∶1000) or goat anti-mouse/rabbit Alexa488 (1∶1000) for 1 hr at room temperature. Sections were washed for 5×10 min with PBST and mounted with Vectashield containing DAPI. Control experiments involved omission of the relevant primary antibody and incubation with the appropriate secondary antibody. Little or no fluorescence was detected in control sections. Antibodies used were as follows: Rabbit anti-ZO-1 (1∶100) and rabbit anti-claudin-1 (1∶60). Rabbit polyclonal anti-keratin 1 and 10 were used at 1∶100 dilution. Secondary goat anti-mouse or goat anti-rabbit Cy3 antibodies were used at 1∶1000 dilution. Images were collected on an Olympus BX51 upright microscope using a 20x/0.30 Plan Fln objective and captured using a Coolsnap HQ camera through MetaVue Software (Molecular Devices). Longitudinal cryosections (8 µm) of tail tendons from 20 day old C57BL/6 mice and, 3 month old *Col4a1* ^+/Svc^ mice and WT littermates were fixed for 10 min in acetone followed by antigen retrieval using 0.1 M HCl and 0.1 M KCl for 10 min. Sections were incubated overnight with the following antibodies: laminin (1∶1000), H22, H31, H69 antibodies against α2(IV) collagen chain, α3(IV) collagen chain and α6(IV) collagen chain (1∶100), nidogen 1 (1∶500) and perlecan (1∶500). Sections were rinsed and incubated with secondary antibody and mounted in Vectashield with Dapi.

Images were captured using a Zeiss LSM510 Meta confocal microscope and Zeiss LSM software. Wax sections of limbs were fixed using a zinc based fixative [12], [13] for 48 hrs at 4°C, followed by 48 hrs in 50% ethanol. The tendons were then filleted off the bone; wax processed using a Tissue-Tek Vacuum infiltration Processor (Bayer Diagnostics, Newbury, UK) and paraffin embedded. Serial sections (7 µm) were obtained. Sections of tendons were dewaxed and treated for antigen retrieval using proteinase K solution (1 mg/ml in 0.1 M Tris-HCl, 50 mM EDTA, pH 8) at 37°C for 30 min. Sections were blocked with 10% normal goat serum in PBS-T for 1 hr at room temperature followed by incubation with laminin antibody (1∶100) in PBS-T with 5% BSA overnight at 4°C. Sections were rinsed for 5×10 min and incubated with goat anti-rabbit Cy3. Sections were washed and mounted using Vectashield containing DAPI.

### Histology

Serial sections (7 µm) were obtained and stained for haematoxylin and eosin. Images were obtained using a brightfield microscope (Axiovision, Zeiss) [5], [12], [13].

### Preparation for electron microscopy

Tendons were prepared for transmission electron microscopy as previously described [14], [15]. Sections (85 nm thick) were collected and examined in a Tecnai 12 BioTwin transmission electron microscope operated at 100 keV accelerating voltage. Images were collected on film at 2900× magnification.

### Isolation of surface cells from e14 chick metatarsal tendon

E14 chick metatarsal tendons were dissected and the surface cells isolated using a variation of the method described by Banes and co-workers to isolate cells from tendon by sequential enzymatic and physical release [16]. The method described here avoids the use of physical scraping of the tendon. In brief, the tendons were incubated in 0.25% trypsin in HBSS (10 ml) for 30 mins at 37°C. The surface cells released by this treatment were collected by centrifugation (the supernatant generated by 1900 rpm for 5 mins in a bench top centrifuge). The tendons were then subsequently digested with 0.5% bacterial collagenase (type 4, Worthington) in 0.25% trypsin in HBSS (6 ml) for 1.5 hours with gentle pipetting every 20 minutes to release the endotenon cells.

### RNA extraction and polymerase chain reaction

Total RNA was isolated from cells using TRIzol reagent followed by DNase treatment. cDNA was transcribed from 2 µg of RNA with TaqMan reverse transcriptase (RT) polymerase, using an oligo(dT)16 primer. RT-PCR analysis was performed using primers complementary to chick β-actin and laminin β1. Amplification of the correctly sized products was verified by electrophoresis on a 2% Trisborate-EDTA gel. The identities of the products were confirmed by DNA sequencing.

### Sequencing of RT-PCR products

Sequencing of PCR products was performed using BigDye Terminator v3.1 cycle sequencing kit. Samples were placed in a thermal cycler under the following conditions: initial denaturation was performed at 96°C for 1 min, followed by 25 cycles of 96°C for 10 seconds, 50°C for 5 seconds, and 60°C for 4 min. Samples were precipitated with ethanol-sodium acetate prior to analysis.

### Protein extraction from mouse deep flexor tendon and western blot analysis

Flexor tendons were dissected from 4 control and 5 Svc mice (5 months old). Tendons were frozen directly in liquid nitrogen and powdered using a Mikro-Dismembrator (2×90 seconds, at 2000 rpm). Tissue was placed into extraction buffer (240 µl) (50 mM Tris-HCl pH 7.4, 150 mM NaCl, 0.5 mM PMSF, 0.5 mM N-ethylmaleimide, 10 mM EDTA) containing an EDTA-free protease inhibitor tablet (1 tablet per 10 ml) (Roche). Samples were rotated overnight at 15 rpm, 4°C. The protein concentration of the extracts was determined using a micro-BCA assay and equal amounts of protein were loaded for each sample and control laminin sample (Engelbreth-Holm-Swarm murine sarcoma, basement membrane) (37.5 µg). Extracts were examined by standard Western blot procedures. Anti-laminin antibody (Sigma Aldrich) and used at 1∶500 dilution.

### Mouse injury model

The mice were anaesthetized as described [17]. Mice were four-week-old C57/BL6. The flexor digitorum profundus in both hind limbs in the mice were exposed by a skin incision. A 50% laceration was made in the tendon between the A1 and A3 pulley. A proximal tenotomy was made at the ankle joint so the tendons were completely divided distally. This immobilized the limb and promoted adhesion development. The skin incisions were closed using 10/0 polyamide sutures (B Braun Medical, Germany). Six mice were euthanized at each time point: day 3, day 21 post-injury. The *Col4a1^+/Svc^* mice were backcrossed for 5 generations on a Bl/6 background and control animals were WT littermates.

## Results

### Tendons have a cell-retentive barrier

**Figure 1:**
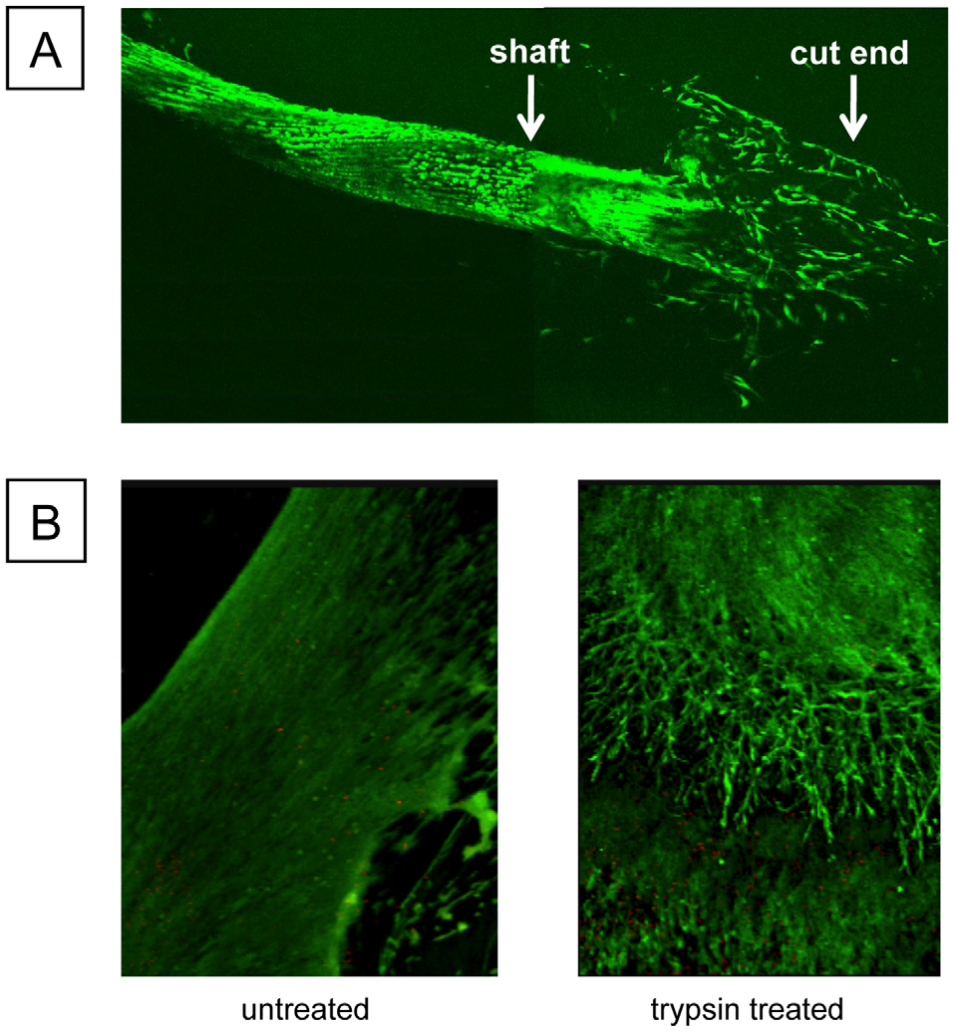
Tendons have a cell-retentive barrier. **A.** *Ex vivo* mouse flexor tendon embedded in a fibrin gel. **B.** *Ex vivo* tendons were briefly subjected to enzymatic treatment (trypsin) and imaged 5 days later. Cells were labeled with Calcein AM (green, living cells) and propidium iodide (red, dead cells).

We hypothesised that a possible mechanism of adhesion formation at the surfaces of injured tendons was the extravagation of cells into the fibrin clot that forms during haemostasis. Therefore, as a first experiment, we incubated *ex vivo* mouse tendons in fibrin and observed the tendon after 5 days in culture by confocal microscopy. Cells migrated from the cut ends but not from the shaft of the tendon (Fig. 1A). In a further experiment we produced a 50% tenotomy (prior to incubation in fibrin) and observed the damaged tendon using time-lapse microscopy. As shown in Movie S1, cells could be seen exiting the injury site. These results suggested that tendons are encapsulated by a cell-retentive layer. To test this hypothesis further, we briefly incubated tendons in trypsin, embedded the tendons in fibrin gels, and assessed cell migration after 5 days. Live and dead cell imaging was preformed with Calcien AM and propidium iodide, respectively. Confocal fluorescence microscopy showed the presence of cells migrating away from the damaged surfaces of the tendons (Fig. 1B). In the trypsin-treated tendons, the edges of the tendon were no longer visible thereby indicating that the surface of the tendon was susceptible to proteolytic damage.

### Identification of a basement-membrane epithelium at the surface of tendon

**Figure 2:**
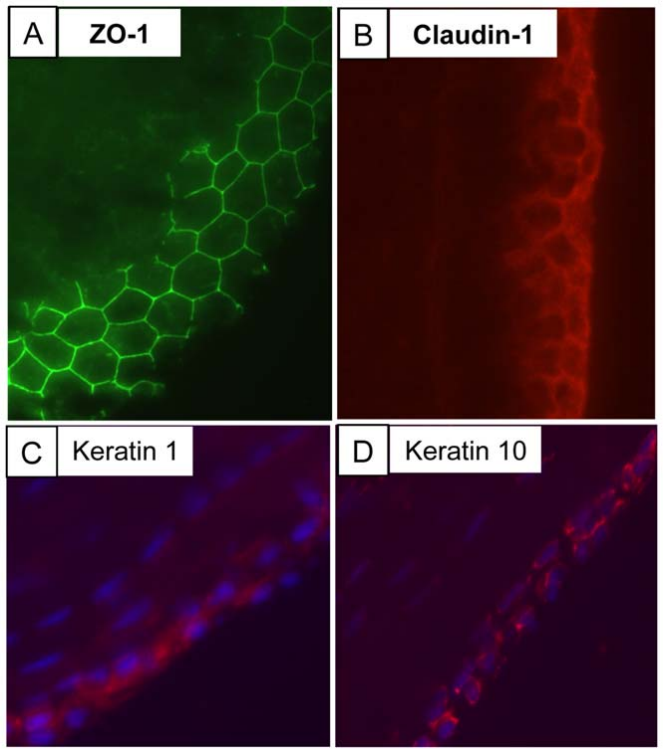
Identification of an epithelium at the surface of tendon. Immunofluorescence detection of ZO-1 (**A**) and claudin-1 (**B**) in transverse sections of day 13 embryonic chick metatarsal tendon; keratin 1 (**C**) and keratin 10 (**D**) in longitudinal sections of postnatal mouse flexor digitorum superficialis. Blue, DAPI staining.

**Figure 3:**
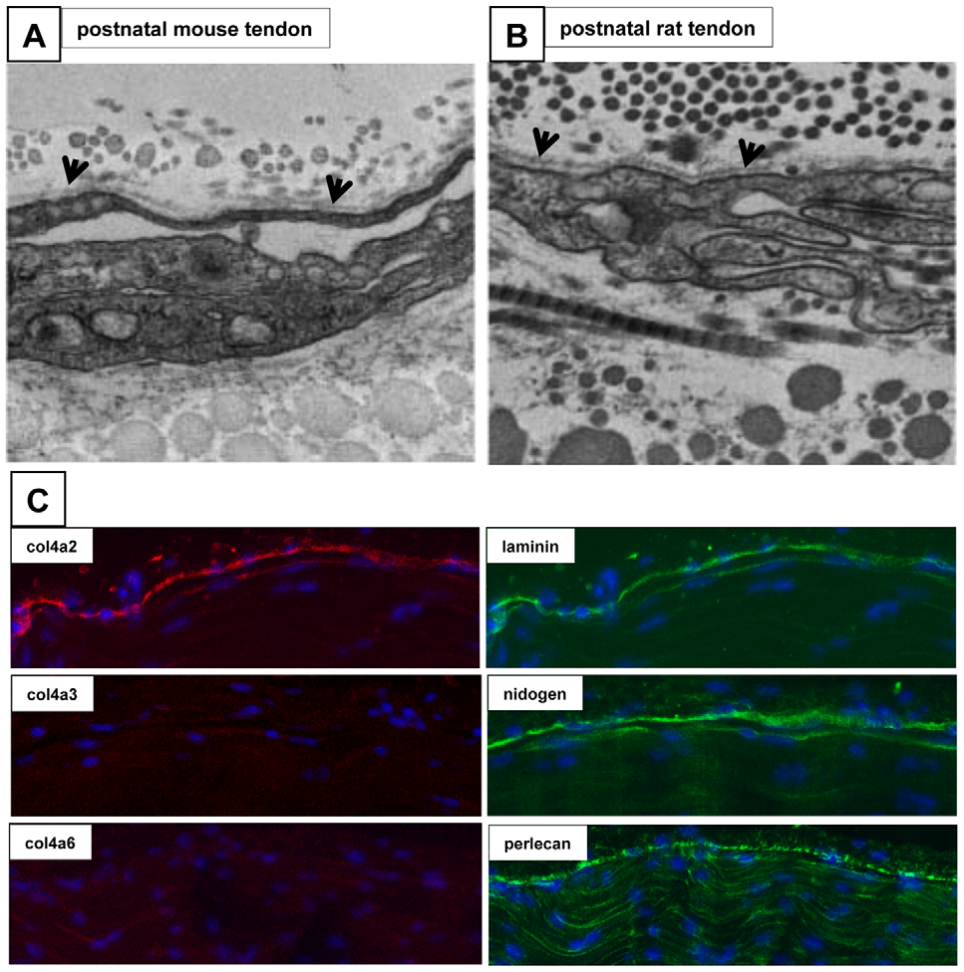
Characterisation of the basement membrane in postnatal mouse and rat tendon. (**A–B**) TEM of mouse and rat tail tendon showing the presence of flat epithelial cells adjacent to a thin BM (arrowheads). (**C**) Immunofluorescence detection of α2(IV) chain of type IV collagen (Col4a2), α3(IV) chain of type IV collagen (Col4a3), and α6(IV) chain of type IV collagen (Col4a6), laminin, nidogen, and perlecan in longitudinal sections of P28 mouse tail tendon.

To learn more about the surface of the tendon, immunofluorescence was performed on embryonic chick metatarsal tendon and mouse flexor tendon (both of which are motion transmitting and load-bearing tendons). The results showed the presence of ZO-1 and claudin-1 tight junctions as well as keratin 1 and 10 (Fig. 2). TEM analysis showed the presence of a BM and epithelial cells with a flattened morphology and interdigitating cell processes, characteristic of epithelial cells in postnatal mouse and rat tendons (Fig. 3). Interestingly, the BM was located on the outermost aspect of the tendon. Immunostaining showed that the BM contains α2(IV) collagen, nidogen-1, laminin, and perlecan (Fig. 3C). The absence of α3(IV) and α5(IV) collagen indicated that the BM contained the α1.α1.α2 network of type IV collagen [18]. While laminin staining was restricted, almost exclusively, to the surface of tendon, perlecan was also detected within the tendon but at apparently lower levels.

### The surface epithelial cells can be isolated by limited proteolysis

**Figure 4:**
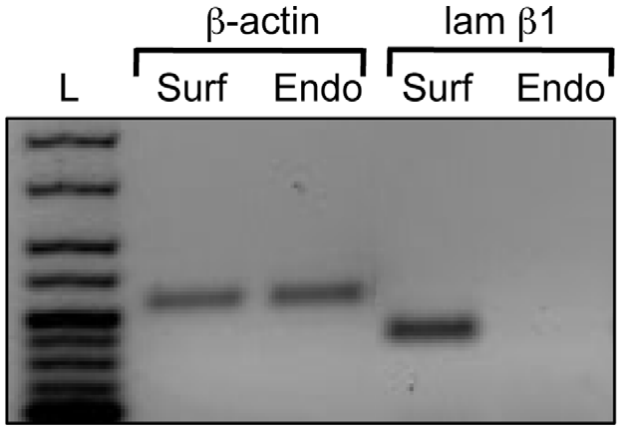
Separation of laminin-expressing cells from the surfaces of tendon. Cells were released from the surface of tendon by limited trypsin digestion. Cells from the central core of the tendon were released by further digestion in trypsin and bacterial collagenase. The presence of laminin β1 was determined by the polymerase chain reaction. The surface (Surf) cells express laminin β1 (lam β1) whereas the endotendon (Endo) cells do not. L, ladder.

The presence of a BM on the outermost aspect of the tendon suggested that limited treatment with trypsin could be effective in releasing the surface cells. Noteworthy, trypsin has limited activity on collagen [19] but is effective at degrading globular proteins such as laminin. Therefore, we incubated *ex vivo* tendons with trypsin and collected the released surface cells by centrifugation. RNA was isolated from the surface (Surf) and endotendon (Endo) cells and examined by reverse transcriptase PCR for the expression of laminin. As shown in Fig. 4, laminin β1 was detected in the Surf but not the Endo preparations.

### A G1064D substitution in the α1(IV) chain of type IV collagen disrupts the tendon basement membrane

**Figure 5:**
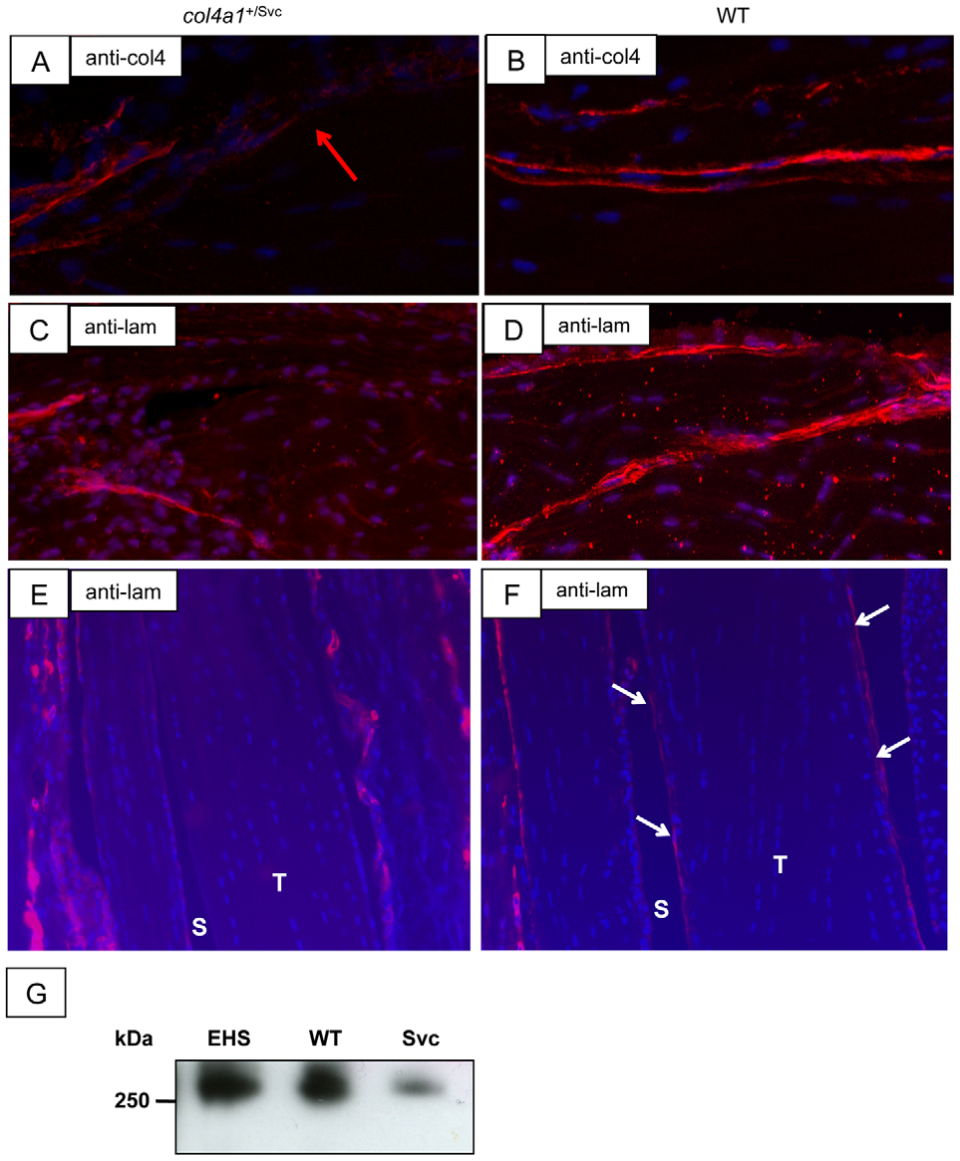
Tail and flexor tendons of the col4^+/SVC^ mouse have a disrupted and interrupted basement membrane. **A**, **C** and **E**, *Col4a1^+/Svc^* mouse samples. **B**, **D** and **F**, wild-type littermate mouse samples. Immunofluoresence detection of collagen IV using an anti-α2(IV) antibody (**A**, **B**) and laminin (**C**, **D**) in longitudinal sections of postnatal mouse tail. (**E**, **F**) Immunolocalisation of lamining in the flexor digitorum profundus in wild-type and Col4a1^+/Svc^ mice. T, tendon. S, synovial space. Blue, DAPI. White arrows, tendon epithelium. Red arrow, discontinuous basement membrane. (**G**), western blot analysis for laminin from the EHS tumor (used as control), and flexor tendons of wild-type (WT) and *Col4a1^+/Sv c^* mice (Svc).

The mechanical integrity of basement membranes depends on an intact type IV collagen network [9]. Having shown that tendon is covered by a BM we wanted to know if a mouse with an abnormal type IV collagen would display a tendon phenotype. We examined the *Col4a1^+/Svc^* mouse that has a G-to-A transition in the *Col4a1* gene producing a G1064D substitution in the α1(IV) chain of type IV collagen. Heterozygous mice have vacuolar cataracts, a small body size, bruising at birth and some arteriolar silvering, which have been attributed to BM defects [20]. The mice are viable, which facilitates analysis of their tendons at postnatal stages of development. Using immunolocalisation we showed that the type IV collagen and laminin staining of the BM in tail tendons of *Col4a1^+/Svc^* mice was discontinuous and disrupted, compared to wild-type littermates (compare Fig. 5A and Fig. 5B). Immunofluorescence using an anti-laminin antibody showed that regions of the flexor tendons of *Col4a1^+/Svc^* mice were devoid of laminin staining (Fig. 5C, E), which was in contrast to littermate controls (Fig. 5D, F). We considered the possibility that the absence of laminin staining in the *Col4a1^+/Svc^* mice might be the result of (i) epitope masking by mutated type IV collagen or (ii) reduced levels of laminin. We performed western blot analysis of protein extracts from flexor tendons of *Col4a1^+/Svc^* and wild-type mice. The results showed a marked reduction in the level of laminin protein in the *Col4a1^+/Svc^* mice compared to wild-type littermates (Fig. 5G). Therefore, the reduced labelling for laminin in the *Col4a1^+/Svc^* mouse samples was most likely the result of reduced levels of laminin protein in the tendon.

### G1064D substitution in the α1(IV) chain leads to spontaneous tendon adhesions

**Figure 6:**
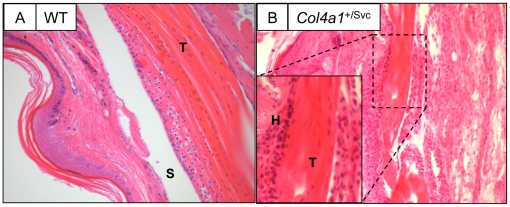
The *Col4a1^+/Svc^* mice have spontaneous tendon adhesions. H & E staining of longitudinal sections through a wild-type (**A**) *and Col4a1^+/Svc^* (**B**) mouse hind limb. Insert shows the absence of a synovial space and hyperplasia (H) of the tendon synovium. Blue, DAPI. S, synovial space.

Histological examination of the deep flexor tendons in the hind limbs of *Col4a1^+/Svc^* mice showed evidence of adhesions (not seen in wild-type mice) between the tendon and the tendon synovium (compare Fig. 6A and 6B). Higher power images of the adhesions show synovial hyperplasia and regions of acellularity in the tendon adjacent to the adhesion (Fig. 6B inset).

### Tenotomy results in tendon adhesions and disruption of the tendon basement membrane

**Figure 7:**
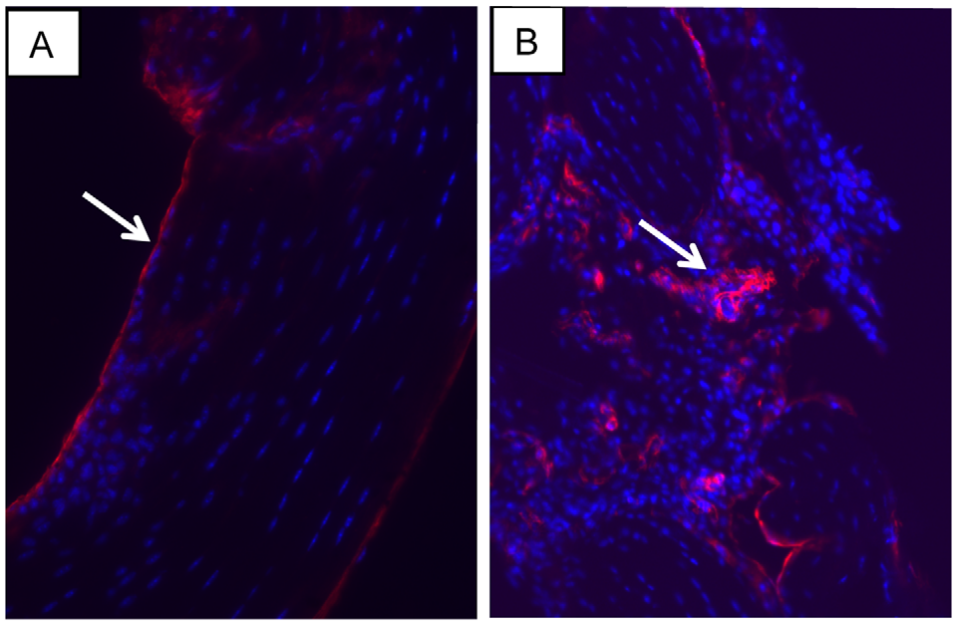
Laminin in tendon adhesions. Immunofluorescence detection of laminin (red) in mouse flexor digitorum profundus 3 days (**A**) and (**B**) 21 days post injury *in vivo*. Blue, DAPI. Arrow, laminin expression at the site of the tenotomy. FDP, flexor digitorum profundus.

As a final experiment, we wanted to know to what extent the tendon basement membrane is disrupted when an adhesion forms at the site of a tenotomy. Under the guidelines of the UK Home Office, we severed the flexor tendons of 4 week-old mice and examined the injury site (in separate animals) for up to 21 days post injury. Examination of the mice showed that adhesions had formed between the tendon and the tendon synovium and skin. The tendon flanking the injury was intact, as shown by the presence of laminin at the intact epithelium (see white arrow of Fig 7A). However, laminin occurred throughout the adhesion (Fig. 7B).

## Discussion

In this study we showed that tendons are covered by a BM-epithelium that prevents cell extravagation and spontaneous formation of fibrous adhesions. We show that the *Col4a1^+/Svc^* mouse, which has a missense mutation in the *Col4a1* gene, has a defective tendon BM and spontaneous adhesions. The results highlight the importance of collagen IV and BMs in normal tendon function.

Our data show that *ex vivo* tendons incubated in fibrin are relatively stable until the surface is damaged (by proteolytic treatment), which leads to cell extravagation into the fibrin gel. Furthermore, rapid cell exit occurs at the cut ends of excised tendons incubated in fibrin gels. These experiments provided the first indication that the cells exit damaged tendons at the site of injury and enter the fibrin-rich environment but are prevented from doing so under physiological conditions by a cell-retentive layer at the tendon surface.

Transmission electron microscopy showed the presence of a distinct BM at the tendon surface. BMs consist of a select group of ECM macromolecules that are constructed around a core of collagen IV and laminin. Early studies identified collagen IV in the endotendineum and endomysium of the *extensor carpi radialis* muscle with attached tendon [21]. Further studies showed the presence of a thin BM-like layer on both sides of ‘flattened fibroblasts’ in the peritendineum of adult rat-tail tendon [22], [23]. Here we showed that a BM is located at the outermost surface of flexor tendons, which corresponds to the peritendineum according to the nomenclature used by Strocchi and co-workers [23]. Immunolocalisation showed that the BM in postnatal tendon contains laminin, perlecan, nidogen-1 and the α1.α1.α2 network of collagen IV. We were able to isolate the surface cells by limited trypsinisation and demonstrate that the surface cells are different from those in the core in that they express laminin β1 as well as other epithelial markers such as keratins (see below).

Previous studies have highlighted the importance of cell-cell junctions in tendon development. The cells in the core of embryonic tendon are connected via adherens junctions that function to organise the cells and influence the parallelism of the tendon matrix [24]. In adult tendon the cells are connected via gap junctions that are involved in intercellular communication in response to mechanical stress [25]. We showed here that the cells at the surface of embryonic tendon are connected by tight junctions, which in other tissues function as a selective permeability barrier, to maintain apicobasal polarity, and regulate cell behaviour (for example see [26], [27], [28]). Staining for tight junction components in embryonic tendon and keratin 1 and 10 in postnatal tendon was a convenient method of visualising the tendon epithelium. Analysis showed that the epithelium in embryonic tendon consisted of several layers of cells whereas in the adult it was a single cell thick. We hypothesise that the epithelium is important for tendon formation during embryogenesis and has a major role postnatally to protect the tendon from adhesion formation.

Our findings of a BM at the tendon surface prompted us to investigate the requirement of collagen IV in tendon development. ENU-induced mutations in mice had generated the Svc mouse that is small and has vacuolar cataracts [29] resulting from a missense mutation in the *Col4a1* gene that encodes collagen IV [20]. Mice that are heterozygous for the mutation (*Col4a1^+/Svc^*) are viable facilitating the examination of flexor tendons of postnatal mice. Examination of 3 month-old *Col4a1^+/Svc^* mice showed that tendons had developed but the BM was interrupted leading to spontaneous formation of adhesions. Immunolocalisation studies of tail and flexor tendon showed that the deposition of laminin and collagen IV was patchy, resulting in regions where the tendon was devoid of BM. Studies of mice deficient for Col4a1 and Col4a2 has shown that collagen IV is required for mechanical stability of postnatal BMs but is dispensable for BM development [9]. Our results confirm that a structural defect in collagen IV can lead to the loss of BM, which is probably most apparent in tissues such as tendon that are under considerable mechanical wear-and-tear. However, the results also show that in the absence of an intact BM, the tendon is susceptible to spontaneous adhesions formation. How the adhesions are formed is unclear, and further studies are needed to investigate the disease mechanism. It is possible that exposure of the fibroblasts underlying the BM to fibrin promotes cell migration. Alternatively, the epithelial cells might undergo epithelial-to-mesenchymal transition and the resultant fibroblasts migrate into the surrounding fibrin-rich environment. In the absence of fibrin (e.g. in the *Col4a1^+/Svc^* mouse), the fibroblasts in the BM-denuded areas of tendon appear to generate opportunistic attachments with overlying structures. However, the formation of adhesions in the absence of blood-vessel in-growth and inflammatory responses (at least in the *Col4a1^+/Svc^* mouse) highlights a new function for the BM-epithelium in preventing adhesion formation.

To investigate further the role of the BM-epithelium in tendon injury we introduced a partial tenotomy in 4 month-old wild-type mice and analysed the tendons for up to 21 days post injury. Intrasynovial adhesions were evident between the tendon and the tendon sheath. At the sites of adhesion formation the tendons were acellular but the sheath displayed mild hyperplasia. These observations are consistent with cells moving out of the injury site where the BM-epithelium is damaged. Laminin was distributed throughout the adhesion and on its surface. Of interest, the presence of an intact BM containing a continuous layer of laminin on the surface of the adhesion would explain the long-term stability of these structures.

In conclusion, the results show the importance of the tendon epithelium in maintaining the functional integrity of the tendon. Furthermore, the results suggest that engineered tendons lacking a BM-epithelium or transplanted tendons with damaged surfaces, might have limited use in tissue engineering and *in vivo* regenerative medicine.

## Supporting Information

Movie S1 **The movie shows time-lapse microscopy of the edge of a tendon that has been partially severed and incubated in a fibrin gel for 5 days.** The injury site is seen as a V-shaped cut at the tendon surface.
(AVI)
